# Development and Characterization of “Green Open-Cell Polyurethane Foams” with Reduced Flammability

**DOI:** 10.3390/ma13235459

**Published:** 2020-11-30

**Authors:** Maria Kurańska, Hynek Beneš, Kamila Sałasińska, Aleksander Prociak, Elżbieta Malewska, Krzysztof Polaczek

**Affiliations:** 1 Department of Chemistry and Technology of Polymers, Cracow University of Technology, Warszawska 24, 31-155 Kraków, Poland; aleksander.prociak@pk.edu.pl (A.P.); elzbieta.malewska@pk.edu.pl (E.M.); krzysztof.polaczek@doktorant.pk.edu.pl (K.P.); 2 Institute of Macromolecular Chemistry, Academy of Sciences of the Czech Republic, V.v.i., Heyrovský Sq. 2, 162 06 Prague 6, Czech Republic; benesh@imc.cas.cz; 3 Department of Chemical, Biological and Aerosol Hazards, Central Institute for Labour Protection—National Research Institute, Czerniakowska 16, 00-701 Warszawa, Poland; kasal@ciop.pl

**Keywords:** open-cell polyurethane foams, bio-polyol, spray foams, flammability, bio-foams

## Abstract

This work presents the cell structure and selected properties of polyurethane (PUR) foams, based on two types of hydroxylated used cooking oil and additionally modified with three different flame retardants. Bio-polyols from municipal waste oil with different chemical structures were obtained by transesterification with triethanolamine (UCO_TEA) and diethylene glycol (UCO_DEG). Next, these bio-polyols were used to prepare open-cell polyurethane foams of very low apparent densities for thermal insulation applications. In order to obtain foams with reduced flammability, the PUR systems were modified with different amounts (10–30 parts per hundred polyol by weight—php) of flame retardants: TCPP (tris(1-chloro-2-propyl)phosphate), TEP (triethyl phosphate), and DMPP (dimethyl propylphosphonate). The flame retardants caused a decrease of the PUR formulations reactivity. The apparent densities of all the foams were comparable in the range 12–15 kg/m^3^. The lowest coefficients of thermal conductivity were measured for the open-cell PUR foams modified with DMPP. The lowest values of heat release rate were found for the foams based on the UCO_TEA and UCO_DEG bio-polyols that were modified with 30 php of DMPP.

## 1. Introduction

Spray polyurethane foams are highly effective thermal insulation materials that can be used both in residential and industrial construction, including internal and external wall insulation such as in ceiling and roofing [1]. The construction of passive and energy-saving houses as well as energy conservation of buildings have boosted the market of spray polyurethane (PUR) foams. The spray PUR market is predicted to double in the next decade and reach $2.5B globally by 2024, with the highest share being in North America (40% of total) [2]. PUR foams are obtained from two components—A and B. Component A comprises polyols, catalysts, surfactants, blowing agents, flame retardants, and other proprietary additives. Component B is a polymeric methylene diphenyl diisocyanate (pMDI), a mixture of 4,4′-methylene diphenyl diisocyanate (4,4′-MDI), other MDI isomers (2,4′- and 2,2′-MDI) and higher oligomers of MDI.

The depletion of petroleum resources is driving the development of polymers and polymer additives from sustainable bio-materials. Natural oils are a promising renewable source for the preparation of polyols due to their renewability, universal availability, and environmental compatibility [3,4]. Thanks to double and ester bonds in their chemical structures, they can be modified to form new polyol structures [5,6].

PUR foams have very good mechanical and physical properties taking into account their low apparent density. Unfortunately, rigid PUR foams, due to their chemical nature and porous structure, are flammable and special attention should be paid to the flammability and toxicity of the gas products emitted during combustion. The flammability of such foams should be limited in order to allow for their applications in building construction [7,8]. Open-cell PUR foams are chemically similar to closed-cell foams, but their combustibility properties differ substantially. The difference comes from their open-cell structure, which offers easier air flow and flame development.

The flammability of PUR foams can be reduced by incorporating flame retardants in the form of liquids or fillers. As far as the application of spray foams is concerned, flame retardants in the liquid form, such as halogen-containing or halogen-free compounds, are preferred. Flame retardants can act in a gas and condensed phase. Halogenated compounds use chemical interference with the radical chain mechanisms in the gas phase during combustion to achieve flame retardancy. In combustion, high energy OH and H radicals are created and scavenged by halogen radicals. Phosphorus-based compounds act in the condensed phase by changing the pyrolytic path of the polymers, also reducing the gaseous combustibles [9].

The use of halogen-containing flame retardants is highly restricted due to the release of large amounts of smoke and toxic gases during combustion [9,10]. However, flame retardants containing halogen are highly effective and still play a major role in reducing the flammability of rigid PUR foams. Most spray PUR formulations contain tris(1-chloro-2-propyl) phosphate (TCPP) as a flame retardant in concentrations of 8–13 wt.%. TCPP is characterized by minimal reactivity and relative low volatility (reported boiling points range from 235 to more than 270 °C) [8,11].

In industry, phosphorus-containing flame retardants have been proposed as alternatives because they produce smaller amounts of toxic combustion products [12].

The aim of this research is to develop non-flammable open-cell PUR foams by employing only bio-polyols from used cooking oil (UCO) as polyol components in the PUR formulation. The influence of TCPP, TEP (triethyl phosphate), and DMPP (dimethyl propylphosphonate)—as commercial flame retardants frequently used in PUR systems—on the properties of open-cell foams, was investigated. The foams were derived from bio-polyols of different chemical structures based on used cooking oil.

The influence of the flame retardants on the thermal properties, compressive strength, cellular structure and apparent density of the modified PUR foams was also examined. The results indicate that the modified used cooking oil is an excellent component for the preparation of non-flammable open-cell PUR foams and this work fits into the idea of a circular economy.

## 2. Materials and Methods

### 2.1. Materials

For the preparation of open-cell PUR foams, two types of bio-polyol obtained by a modification of UCO were applied. UCO was transesterified with triethanolamine (UCO_TEA) and diethylene glycol (UCO_DEG). Bio-polyols were synthesized at Cracow University of Technology. Selected properties of both polyols are shown in Table 1. Polymeric methylene diphenyl diisocyanate with the trade name Ongronat 2100 was supplied by Borschodchem (Kazincbarcika, Hungary) The average functionality of this component was 2.6 to 2.7. The carbon dioxide generated in the reaction of water and isocyanate groups was used as a chemical blowing agent. Catalysts of the foaming and gelling processes as well as a surfactant were provided by Evonik (Essen, Germany). TCPP, TEP, and DMPP from LANXESS AG (Cologne, Germany) were added in order to reduce the flammability of the PUR foams. Selected properties of the flame retardants according to the technical data sheet are shown in Table 2.

### 2.2. Preparation of Open-Cell PUR Foams

Open-cell PUR foams were prepared on a laboratory scale using a one-step method from two-component (A and B) systems. The influence of the content and type of the flame retardants on the processing of the PUR system and the properties of the resulting porous materials were investigated. Three contents of the flame retardant were applied: 10, 20, and 30 php (parts per hundred polyol by weight). The open-cell PUR foams based on bio-polyol UCO_TEA and UCO_DEG (100 php) were used as reference materials. Component A consisted of bio-polyol (UCO_TEA or UCO_DEG) 100 php, catalysts, surfactant, flame retardant, and blowing agent. Component B was isocyanate. The isocyanate index (the ratio of isocyanate groups to hydroxyl groups in a reaction mixture) of the final materials was 1. The PUR formulations derived from each bio-polyol used in the experiment are shown in Table 3 and Table 4. Components A and B were mixed with a mechanical stirrer and then poured into an open mold. The foams were conditioned at room temperature for 24 h and cut into samples appropriate for testing.

### 2.3. Characterization of Foaming Process and PUR Foams Properties

The foaming process was analyzed using FOAMAT equipment. A detailed description of the analysis method was presented in our earlier publication [13]. The device can record changes in the temperature, dielectric polarization, pressure, and rise velocity of reaction mixtures. The morphology of cells was analyzed using a scanning electron microscope TM3000 (Hitachi, Tokyo, Japan) and the software ImageJ (version 1.53f, U. S. National Institutes of Health, Bethesda, MD, USA) was used for image analysis. Anisotropy was calculated as the ratio: major_axis/minor_axis. The closed-cell content in the foams was measured according to the ISO 4590:2016 standard [14]. The apparent density of the samples was calculated in accordance with ISO 845:2006 [15] as a ratio of the sample weight to the sample volume. Thermal conductivity was measured using a heat flow meter instrument Fox200 (TA Instruments, New Castle, DE, USA) and foam samples with dimensions of 5 cm × 20 cm × 20 cm (ISO 8301:1991) [16]. The thermal conductivity value, at an average temperature of 0 °C, 10 °C, and 20 °C, was measured at the rate of steady state heat transfer across a foam with a known thickness. The heat transfer was induced by two different known temperatures between two opposite surfaces of the foam. In this case, one plate of the instrument was set to 0 °C and the other to 20 °C. Compression tests of the foams were conducted according to PN-EN 826:2013-07 [17] in two directions: parallel and perpendicular to the direction of foam rise. The compressive strength was measured at 10% deformation using a Zwick 1445 instrument (Zwick Roell Group, Ulm, Germany). The compressive force was applied at a speed of 2 mm/s, axially in a perpendicular direction to the square surface. The mechanical properties of the foams were evaluated in two directions; parallel and perpendicular to the foam rise direction. Burning behavior was assessed using a cone calorimeter from Fire Testing Technology (East Grinstead, United Kingdom). The samples (100 mm × 100 mm × 25 mm) were placed in aluminum foil and tested at an applied heat flux of 35 kW/m^2^, following ISO 5660-1:2015 [18]. Separation space between samples and the heater was set at 25 mm. Spark ignition was used to ignite the pyrolysis products. After tests, the residues were photographed using a digital camera; an EOS 400 D from Canon Inc. The oxygen index (LOI) was determined according to ISO 4589-2:2017 [19]. Thermogravimetric analysis-Fourier transform infrared spectroscopy (TGA-FTIR) was performed using Pyris 1 TGA analyzer (Perkin Elmer, Waltham, MA, USA) coupled with infrared spectrometer Spectrum 100T FT-IR (Perkin Elmer, Waltham, MA, USA) through a transfer line TL 8000 (Perkin Elmer, Waltham, MA, USA). Samples (ca. 5 mg) were heated from 30 to 800 °C with heating rate of 10 °C/min under a nitrogen flow of 25 mL/min. The optical cell of FTIR spectrometer and the transfer line between TGA and FTIR were heated at 260 °C and 250 °C, respectively, to prevent condensation of evolved gases. FTIR spectra were continuously recorded during the whole TGA run in the wavenumber range 650–4000 cm^−1^ over two scans with a resolution of 4 cm^−1^. H_2_O/CO_2_ software correction (TimeBase, Perkin Elmer, Waltham, MA, USA) was always applied during TGA-FTIR analysis. Temperature of 5% and 10% weight loss (Td 5% and Td 10%) temperature of maximum decomposition (Td max) and char yield at 750 °C were evaluated for all samples. Standard deviation of the TGA measurement was under 5%.

## 3. Results and Discussion

**Figure 1:**
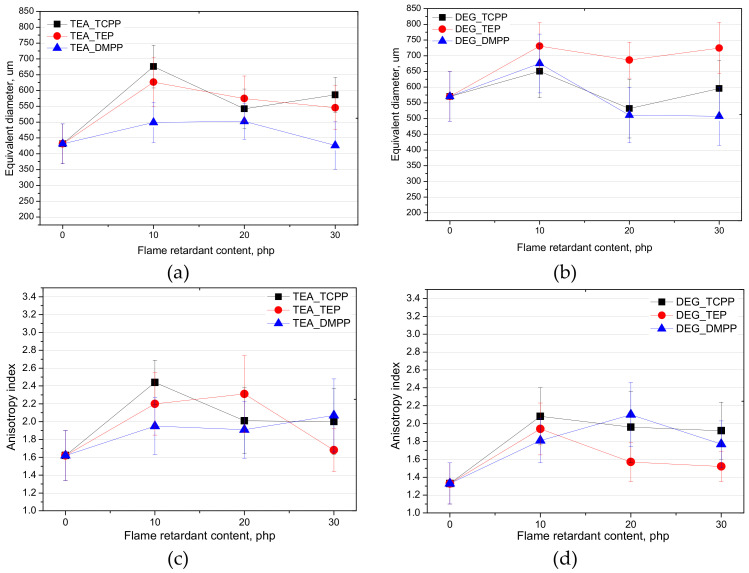
Influence of flame retardant type and content on equivalent diameter (**a**,**b**), anisotropy index (**c**,**d**) of bio-foams based on bio-polyol UCO_TEA and UCO_DEG, respectively.

**Figure 2:**
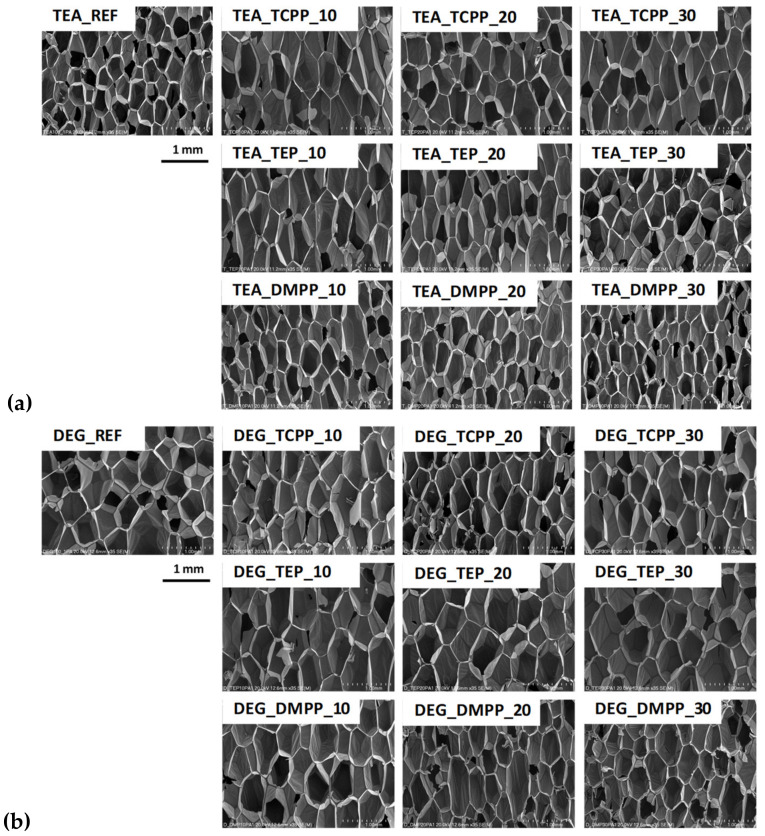
Scanning electron microscopy images of cellular structure of bio-foams based on UCO_TEA (**a**) and UCO_DEG (**b**).

The microstructure and apparent density of foams affect their physical and mechanical properties. When the foam cells are closed, the porous materials behave similar to solids, leading to a rise in the compressive strength [20]. In the case of foams with a low apparent density (<15 kg/m^3^) and open cells, investigating into the effect of additives, such as a flame retardant on the cell structure, is particularly important due to a very low volume of the PUR matrix. The influence of the commercial flame-retardant type and content on the equivalent diameter and anisotropy index of the open-cell PUR foams prepared using bio-polyols UCO_TEA and UCO_DEG, is shown in Figure 1. SEM images of the foams are shown in Figure 2.

**Figure 3:**
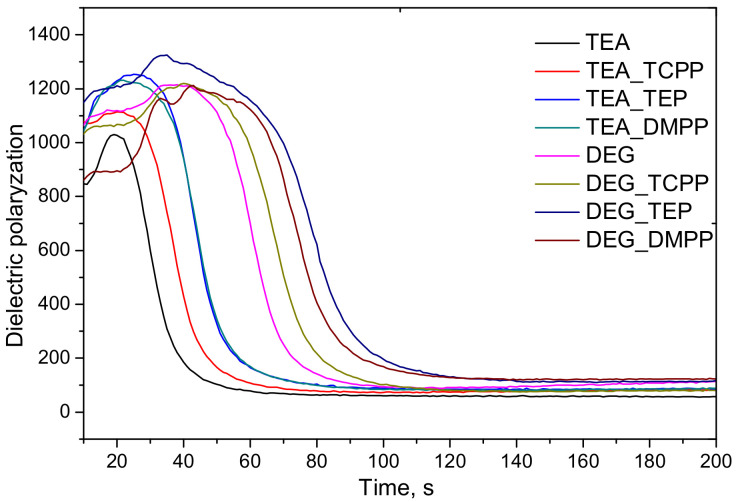
Dielectric polarization of the PUR system modified with a flame retardant (30 php).

It was observed that the bio-foams based on bio-polyol UCO_TEA were characterized by a lower equivalent diameter compared to this parameter measured for the bio-foams prepared based on bio-polyol UCO_DEG. Regardless of the type of flame retardant, the introduction of 10% of this additive increased the diameter of the cells in the foams. It is known that additive flame retardants have a plasticizing effect on the polymer matrix and can also act as surfactants. In this case, the plasticization of the matrix could cause an increase in the size of the cells. The larger diameter of DEG_REF cells can be an effect of lower viscosity and hydroxyl value of bio-polyol UCO_DEG. Such properties can have an influence on a decrease of the DEG_REF system reactivity and consequently cell formation during the foaming process. It was noticed that the type of flame retardants had an influence on the cell diameters in the foams. The lowest value of this quantity was obtained for the materials modified with DMPP. However, the final cell structure of porous materials depends on the curing reaction and network formation [20]. Faster polymerization is known to yield foams with a smaller average cell size [21]. Lower reactivity of the DEG_REF system was confirmed by changes of the dielectric polarization (Figure 3). Figure 3 shows the changes in the dielectric polarization of the PUR reaction mixtures, without a flame retardant (TEA_REF and DEG_REF) and also modified with different flame retardants.

The open-cell bio-foams based on bio-polyol UCO_TEA exhibited the fastest decrease in the dielectric polarization. Dielectric polarization decreases with the reaction progress [13]. This property was measured in a cylindrical container placed on a device containing a built-in dielectric polarization sensor. This sensor was made of two comb-shaped electrodes forming a plane capacitor integrated with a pressure measurement device located in the base plate on which the polyurethane system is poured. The expanding foam provides close contact with the sensor, which ensures direct penetration of the electric fringe field. Changes in the dielectric polarization are directly correlated with the capacity of the sensor. The dielectric polarization is produced by molecules with a large dipole moment due to their polar ends (OH groups of polyols, NCO groups of isocyanates). The reaction between OH and NCO groups, PUR chains formation and cross-linking reaction ultimately suppress dipole mobility. The final curing of the foam is visible as a low and constant signal. The difference in the changes of the dielectric polarization for the PUR systems tested can be associated with the higher hydroxyl value of UCO_TEA comparing to UCO_DEG. What is more, the presence of free electron pairs in nitrogen atoms makes UCO_TEA act as a catalyst. A similar effect was observed in our earlier work investigating closed-cell PUR foams. The formulations modified with the bio-polyol containing amine groups were more reactive and exhibited a faster decrease of the dielectric polarization than the formulations based on the bio-polyol obtained in the epoxidation and ring-opening reactions [22]. The modification of the PUR formulations with flame retardants decreased the reactivity of the systems. This effect can be associated with a dilution of the reaction mixture by the flame retardants as non-reactive components of low viscosity.

**Figure 4:**
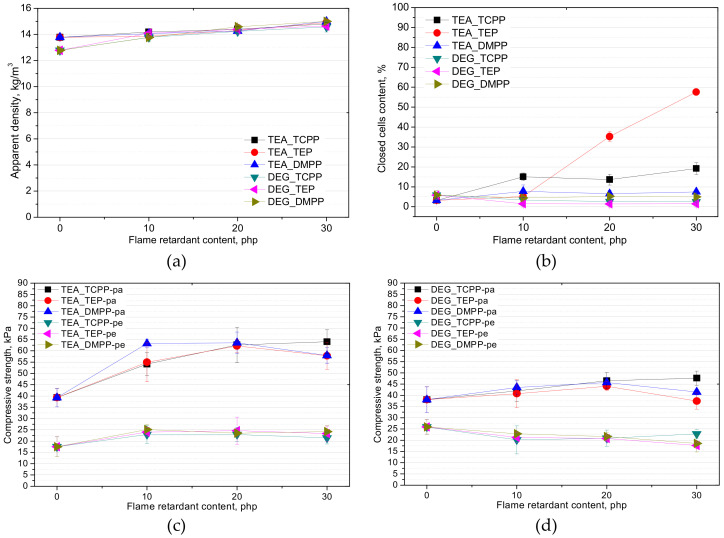
Apparent density (**a**), closed cells content (**b**), and compressive strength (**c**,**d**) of foams based on bio-polyols UCO_TEA and UCO_DEG.

The apparent density and closed cell content of foams has an influence on their mechanical and heat insulating properties [23]. The influence of the chemical structure of bio-polyol and the type, as well as content of flame retardant on the apparent density, closed cell content, and compressive strength of the foams studied in this work, is shown in Figure 4.

The open-cell PUR foams were characterized by the apparent density in the range 12.5–15 kg/m^3^. It was noticed that a modification of the foams with flame retardants caused an increase of the apparent density regardless of bio-polyol type. However, a slightly stronger effect of apparent density rise was observed in the case of the bio-foams based on bio-polyol UCO_DEG during the first phase of foam modification with a flame retardant in an amount of 10 php. The effect can be connected with lower reactivity of this PUR system.

The modification of the TEA_REF system with the flame retardant TEP caused an unexpected increase of the closed cell content from 3% to 58% (Figure 4b). This effect can be associated with the plasticization of the PUR matrix (higher elasticity) by TEP and the higher reactivity of this system, which reduced the effect of wall breaking, while it was not observed in the case of the DEG_REF foam. The foams based on bio-polyol UCO_DEG were characterized by a closed cell content lower than 6% regardless of the type of flame retardant.

Despite similar changes of the apparent density of the bio-foams modified with different flame retardants, higher compressive strength was obtained for the materials based on bio-polyol UCO_TEA. The compressive strength was measured in two directions: perpendicular (pe) and parallel (pa) to the foaming direction of the PUR systems. This is advisable, since the foam cells have a tendency to be elongated in the direction of foam free rise. The values of this parameter in parallel direction were in the range 40–60 kPa for the materials modified with UCO_TEA and 40–45 kPa for the foams based on UCO_DEG. In the case of perpendicular direction, values of compressive strength were lower. However, differences between systems based on UCO_TEA and UCO_DEG were not significant. This effect can be associated with the cellular structure as well as chemical structure of the foams. The foams based on UCO_TEA were characterized by smaller cells than the foams based on UCO_DEG. As shown in Figure 1a,b, the changes in the cell diameter of the foams had no effect on the compressive strength value (Figure 4b,c). This is an interesting result because in the case of foams with higher apparent densities >35 kg/m^3^ the size of cells determines the compressive strength by distributing the compressive stresses over the more numerous structures present in small cells to avoid concentrating the stresses onto fewer larger cell structures. The smaller the cell size in a PUR structure, the higher the value of the compressive strength is. It can be concluded that in the case of very-low-density foams (<15 kg/m^3^) such a relationship does not occur. The higher compressive strength of the foams based on bio-polyol UCO_TEA can also be an effect of their higher hydroxyl value and consequently higher amounts of hard segments in the structures of the PUR matrices.

Both open-cell and closed-cell foams are mainly used as thermal insulation materials. The standard thermal conductivity is given for an average measurement temperature of 10 °C and the data for other conditions are rarely presented. We decided to provide the values of thermal conductivity at different average measurement temperatures because it is important from an industrial point of view. Foam materials are used at different temperatures depending on the application, which affects the effectiveness of heat insulation. In the case of open-cell PUR foams, the coefficient of thermal conductivity is much higher compared to closed-cell materials. This is a result of the open-cell structure, which has an influence on the thermal conductivity [0.037–0.039 W/(m·K)] due to the replacement of blowing agents with air. The thermal conductivity of closed-cell foams is in the range 0.018–0.030 W/(m·K) and depends on the gas composition in closed cells [24,25]. Moreover, the thermal insulating properties of cellular materials are dependent on their cellular structures and closed cells content. The smaller the size of cells in a PUR structure, the lower the thermal conductivity becomes. In this work, the TEA_REF foam was characterized by a lower coefficient of thermal conductivity than the DEG_REF foam, regardless of the average measurement temperature (Table 5). This can be a result of a lower value of the equivalent diameter of cells. Heat transfer can be minimized by reducing the cell size of foams according to heat transfer theory [24]. A confirmation of the beneficial effect of smaller cells is also the values of the thermal conductivity coefficients of the open-cell PUR foams modified with DMPP. Regardless of the type of bio-polyol, the foams modified with this flame retardant were characterized by the lowest values of the heat conduction coefficient. Usually, closed-cell foams have lower heat conductivity. However, this effect was not observed for the materials based on UCO_TEA modified with TEP, which were characterized by a higher content of closed cells compared to the other foams (Figure 4b). This difference may be related to the fact that foams with very low densities have very thin walls and the diffusion of carbon dioxide is very fast. Moreover, in the case of such foams the heat transfer by radiation is also increased. This may explain why the effect of lower thermal conductivity was not observed.

The values of the thermal conductivity are comparable to those obtained in our earlier work, where the foams had different proportions of bio-polyol having used the epoxidation method and opening of oxirane rings. For comparison, the material obtained in 100% from petrochemical polyol was characterized by a value of the thermal conductivity coefficient of 45.76 mW/(m·K) [26].

**Figure 5:**
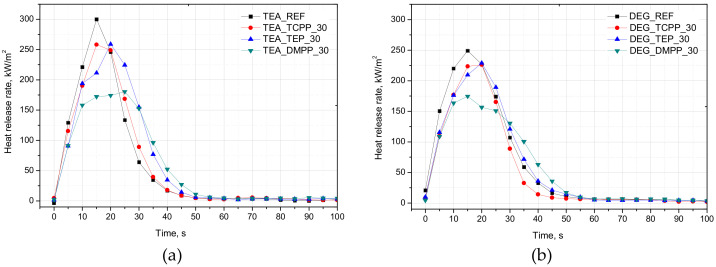
Representative curves of heat release rate curves of TEA (**a**) and DEG (**b**) series.

The PUR foams were examined using a cone calorimeter, which simulates fire development. Table 6 presents the average values of fire hazard parameters, while Figure 5 shows the most representative heat release rate (HRR) curves, delivering information on the burning behavior of the materials.

As can be seen in Table 5, the samples of the PUR foams ignited after at least 2 s and burned for approximately another 42 s. Only in the case of DEG_TEP_30 and the PURs with DMPP was the time to flameout (TTF) a bit longer compared to the other samples. As shown in Figure 5, the TEA foam was burning intensively, and the peak heat release rate (pHRR) reached 297 kW/m^2^. In comparison, the pHRR values of TEA_TEP_30 and TEA_DMPP_30 were reduced by 13% and 39%, respectively. In the case of TEA_TCPP_30, the addition of 30 php of TCPP decreased pHRR by 15%, which meant that the halogen fire retardant used was equally (TEP) or less (DMPP) effective in terms of increasing the fire retardancy of PURs than organophosphorus compounds. The lowest values of pHRR in the case of the DEG series were also found for the foam modified with DMPP (31% decrease). Considering the uncertainties, it can be concluded that the differences between the results were small, and a significant decrease in HRR was observed only when 30 php of DMPP was added. Furthermore, both PURs with DMPP showed the lowest values of the maximum average rate of heat emission (MARHE), which, given the ease of ignition and heat release rate, informs us about the fire hazard posed by the material.

The total heat release, representing a measurement for the fire load of material for the unmodified foams was 7 MJ/m^2^, while for TEA_REF and DEG_REF, with fire retardants, it was decreased by approximately 15% (with the exception of DEG_TEP_30). Despite a significant reduction in the heat release rate of PURs with DMPP, a longer time to flameout led to relatively high THR values [27,28]. On the other hand, the decrease in the effective heat of combustion (EHC) in the case of the DEG series suggests an inhibition of free radicals emission. The most favorable results were obtained for DEG_TCPP_30 and DEG_DMPP_30, where EHC was 24% and 19% lower compared to the unmodified PUR, respectively. Halogen-based fire retardants, such as TCPP, use chemical interference with the radical chain mechanisms in the gas phase [29]. Similarly, DMPP, by decomposing to gaseous PO_2_ fragments, may inhibit the free radical chain reaction. The reduced fire load, besides lower combustion efficiency, can also be caused by char formation. The yield of residues in the case of TEA and DEG modified with fire retardants reached 6–12% and 11–16%, respectively.

Polymers usually emit smoke and toxic gases while burning. Therefore, the total smoke release (TSR) is an important parameter used to evaluate the fire hazard of the [30]. In our work, the addition of the flame retardants under investigation resulted in an inconsiderable increase in smoke emissions. As shown in Table 5, the lowest TSR from TEAs containing fire retardant were obtained for TEA_DMPP_30, and the value was similar to that of the unmodified PUR. In turn, in the case of the remaining samples, the increase reached approximately 9%. For DEG_REF with fire retardants, TSR was 20 to 24% higher compared to unmodified PUR regardless of the substance used. Usually, when fire retardants are active in the gas phase, leading to incomplete combustion, an increase in smoke emission is observed [10].

Limiting oxygen index (LOI) is often used to evaluate the flammability of materials [31]. The LOI values of bio-foams increased for materials modified with flame retardants achieving values higher than 21%, regardless of their type.

**Figure 6:**
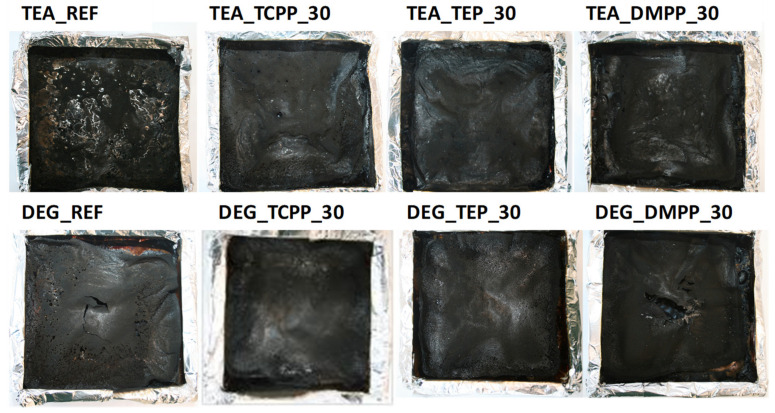
Photographs of samples after cone calorimetry tests.

**Figure 7:**
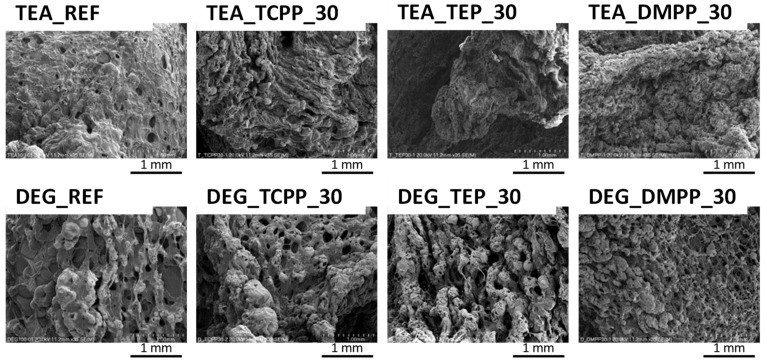
SEM images of char residues.

Digital photographs of the bio-foams after CC tests and SEM photos of char residues are presented in Figure 6 and Figure 7, respectively.

TEA_REF produced a residue in the form of a very thin layer with large holes. TEA_TCPP_30 exhibited a more compact structure with a lot of holes, while TEA_TEP_30 and TEA_DMPP_30 displayed homogeneous but still quite thin char layers. Thin char layers were also formed in the case of the DEG series. Moreover, the char layers have either numerous small holes or a few large ones depending on the flame retardant mechanism of the substances. The phosphorus-based compounds usually act in the condensed phase by changing the pyrolytic path of the materials and reducing the gaseous combustibles [29]. Lorenzetti et al. [32], by examining PUR foams modified with DMPP and TEP, observed that the fire retardants completely volatilized into the gas phase.

**Figure 8:**
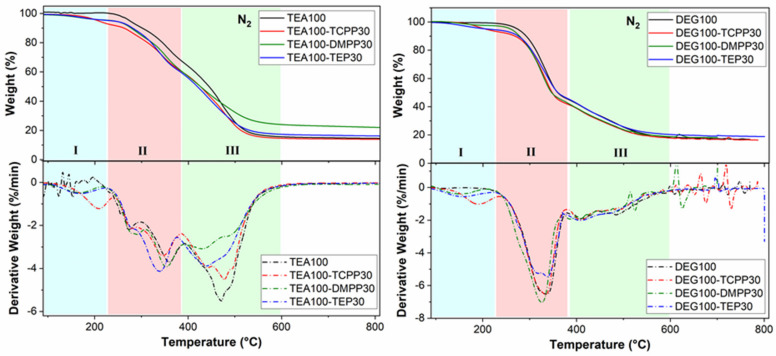
TG and DTG curves (in nitrogen atmosphere) of TEA and DEG series modified with fire retardants.

**Figure 9:**
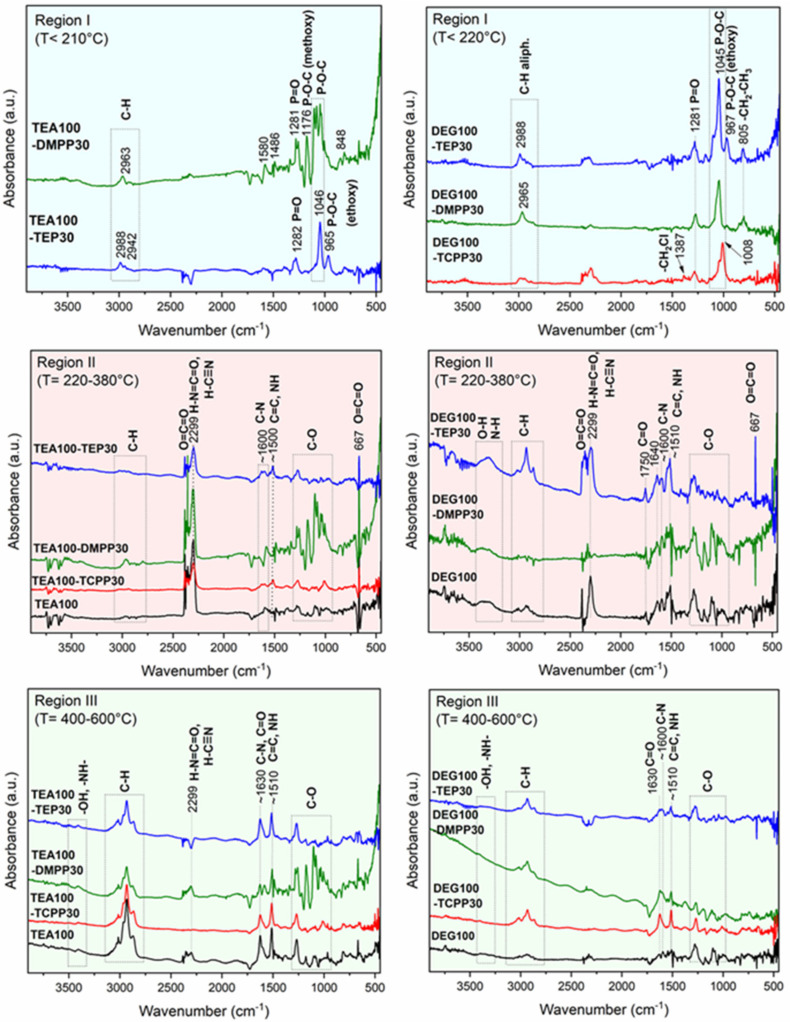
FTIR spectra of volatile products during the non-oxidative thermal degradation of TEA and DEG series modified with fire retardants.

Thermal stability of the PUR foams under an inert (nitrogen) atmosphere was investigated by TGA-FTIR (Table 7, Figure 8 and Figure 9). The TGA results showed that the addition of all fire retardants slightly decreased the thermal stability and the initial stage of degradation of the PUR due to the volatilization of flame retardants (Region I, T_d max1_ in Table 6). Two following degradation steps (Regions II and III) connected to the polymer structure decomposition were also slightly shifted to lower temperatures (T_d max2_ and T_d max3_ in Table 6). The char residue content after TGA run (at 750 °C) was similar to that of the reference foam in the cases of the TCPP-modified foams, higher for the TEP-modified foams and the highest for the DMPP-modified foams, which correlated well with the results from cone calorimetry.

The TG ad DTG curves of the TEA and DEG foams in nitrogen atmosphere were similar to each other and showed three mass losses (Figure 8). The first mass loss occurred at T < 220 °C (Region I), and is connected to volatilization of the flame retardants (as also proved by the FTIR spectra of the evolved gases in Region I, Figure 9) [32,33,34] and, therefore, is not visible for the reference (fire retardant-free) PUR foams. The absorption bands of the P=O (~1280 cm^−1^) and P-O-C (1000–1100 cm^−1^) stretching vibrations are clearly seen, proving the volatilization of phosphorus-containing fire retardants. Additionally, the absorption bands of P-O-C ethoxy (965 cm^−1^), P-O-C methoxy (1176 cm^−1^) and CH_2_-Cl (1387 cm^−1^) directly confirm volatilization of TEP, DMPP and TCPP, respectively.

During the second weight loss (T = 220–380 °C, Region II in Figure 8), the thermal degradation of polymer network of PUR foams proceeded. The FTIR spectra, at maximum decomposition rate in Region II (Figure 9), detected beside the dominant absorption bands (partially suppressed applying the H_2_O/CO_2_ correction) of water (~3600 and 1650 cm^−1^) and CO_2_ (~2350 and 667 cm^−1^), the bands around 2800 to 3050 cm^−1^ (attributed to C-H and =C-H bands of saturated and unsaturated hydrocarbons), at 2299 cm^−1^ (assigned to isocyanates and nitriles), at 1750 cm^−1^ (attributed to C=O of urethanes and esters), at ~1600 cm^−1^ (attributed to C=O of aldehydes and ketones), at ~1500 cm^−1^ (attributed to N-H stretching of amide and C=C stretching of aromatic groups) and around 1000 to 1300 cm^−1^ (assigned to ethers) [33,35]. Furthermore, it is seen that the degradation of PUR foams in Region II was more intensive for DEG-foams (weight loss of ca. 50 wt.%) than for TEA-foams (weight loss of ca. 30 wt.%) as also indicated by the presence of the intensive FTIR bands of C=O (1750 cm^−1^) and C-H (2800–3050 cm^−1^) vibrations for the DEG-foam series. DEG-foams were thus more thermally labile than TEA-foams, which corresponded well to the less cross-linked structure of DEG-foams compared to TEA-foams. Similarly, the thermal stability of the UCO_DEG polyol segments was most likely lower than in the case of the UCO_TEA segments.

In contrast, the FTIR bands of the C=O, C-N (~1630 cm^−1^) and C-H (2800–3050 cm^−1^) vibrations were dominant during the degradation of TEA-foams in Region III at T = 400–600 °C (Figure 8 and Figure 9) evidencing the degradation of the UCO_TEA polyol segments. Moreover, during this weight loss, the residual PUR structure as well as the formed polymeric fragments were further pyrolyzed. The FTIR spectra of the evolved gasses detected the absorption bands of unsaturated and saturated hydrocarbons (2800–3100 cm^−1^), aromatics (1510 cm^−1^), amine derivatives (~3400, ~1600, and 1510 cm^−1^) and ethers (1000–1300 cm^−1^), while the absorption bands of CO_2_, water, isocyanates and nitriles almost completely disappeared [33]. These findings are in good agreement with previous studies showing the formation of aromatic amines (derived from anilines) and random polyol chain scission [33,36].

## 4. Conclusions

Two types of bio-polyols synthesized from used cooking oil and three different flame retardants were applied in preparation of open-cell polyurethane foams with apparent densities of ca. 13 kg/m^3^ and reduced flammability. The modification of the PUR formulations with the flame retardants caused a decrease of the system reactivity. The system based on the UCO_TEA bio-polyol was characterized by higher reactivity compared to that with the UCO_DEG bio-polyol. Synthesis of open-cell PUR foams based only on bio-polyols from used cooking oil is in line with a circular economy as well as green chemistry.

The smallest changes in the PUR system reactivity were observed when using the DMPP flame retardant. This retardant allowed obtaining foams with the smallest cells, the most favorable thermal conductivity coefficient, and the best flame retardancy. The limiting oxygen indices of the bio-foams increased for the materials modified with the flame retardants reaching values higher than 21%, regardless of their type. The thermal stability of the modified foams was slightly lower due to flame retardant evaporation.

## Figures and Tables

**Table 1:** Selected properties of bio-polyols.

Bio-Polyol	Hval [mgKOH/g]	Aval [mgKOH/g]	Mn, [g/mol]	f	D	Ƞ, [mPa·s]	d, [g/cm^3^]	H_2_O, [wt.%]
UCO_DEG	277	1.00	381	1.88	1.29	56	0.958	0.05
UCO_TEA	348	2.31	357	2.21	1.46	182	0.975	0.05

UCO_DEG – bio-polyol obtained by transesterification of used cooking oil method with diethanolamine; UCO_TEA – bio-polyol obtained by transesterification of used cooking oil method with triethanolamine; Hval—hydroxyl value; Aval—acid value; Mn—number molecular weight; f—functionality; D—dispersity; Ƞ—viscosity; d—density; H_2_O—content of water.

**Table 2:** Selected properties of flame retardants.

Properties/Flame Retardant Symbol	TCPP	TEP	DMPP
Phosphorus content, %	9.5	17.0	20.3
Chlorine content, %	32.5	-	-
Acid value, mgKOH/g	<0.05	<0.05	<0.05
Density, g/cm^3^	1.289	1.065–1.074	1.072
Boiling point, °C	270	215	185
Viscosity, mPa·s	75	1.7	2.5

**Table 3:** Formulations of PUR foams based on bio-polyol UCO_TEA.

Component [g]	TEA_REF	TEA_TCPP_10	TEA_TEP_10	TEA_DMPP_10	TEA_TCPP_20	TEA_TEP_20	TEA_DMPP_20	TEA_TCPP_30	TEA_TEP_30	TEA_DMPP_30
UCO_TEA	100	100	100	100	100	100	100	100	100	100
Polycat 218	4	4	4	4	4	4	4	4	4	4
Tegostab B 8870	4.5	4.5	4.5	4.5	4.5	4.5	4.5	4.5	4.5	4.5
Ortegol 500	0.5	0.5	0.5	0.5	0.5	0.5	0.5	0.5	0.5	0.5
Water	15	15	15	15	15	15	15	15	15	15
TCPP	0	10	0	0	20	0	0	30	0	0
TEP	0	0	10	0	0	20	0	0	30	0
DMPP	0	0	0	10	0	0	20	0	0	30
Ongronat 2100	312	312	312	312	312	312	312	312	312	312
%UCO_TEA *	22.9	22.4	22.4	22.4	21.9	21.9	21.9	21.5	21.5	21.5

* Bio-polyol UCO_TEA content in PUR foam.

**Table 4:** Formulations of PUR foams based on bio-polyol UCO_DEG.

Component [g]	DEG_REF	DEG _TCPP_10	DEG _TEP_10	DEG _DMPP_10	DEG _TCPP_20	DEG _TEP_20	DEG _DMPP_20	DEG _TCPP_30	DEG _TEP_30	DEG _DMPP_30
UCO_DEG	100	100	100	100	100	100	100	100	100	100
Polycat 218	4	4	4	4	4	4	4	4	4	4
Tegostab B 8870	4.5	4.5	4.5	4.5	4.5	4.5	4.5	4.5	4.5	4.5
Ortegol 500	0.5	0.5	0.5	0.5	0.5	0.5	0.5	0.5	0.5	0.5
Water	15	15	15	15	15	15	15	15	15	15
TCPP	0	10	0	0	20	0	0	30	0	0
TEP	0	0	10	0	0	20	0	0	30	0
DMPP	0	0	0	10	0	0	20	0	0	30
Ongronat 2100	289	289	289	289	289	289	289	289	289	289
%UCO_DEG **	24.2	23.6	23.6	23.6	23.1	23.1	23.1	22.6	22.6	22.6

** Bio-polyol UCO_DEG content in PUR foam.

**Table 5:** Coefficient of thermal conductivity and closed cell content of PUR foams.

Symbol	Coefficient of Thermal Conductivity [mW/m·K]					
	−10 °C/10 °C		0 °C/20 °C		10 °C/30 °C	
TEA_REF	37.15	±1.41	39.06	±1.31	40.44	±1.22
TEA_TCPP_10	39.03	±0.61	41.01	±0.58	43.26	±0.43
TEA_TCPP_20	38.50	±0.69	40.41	±0.49	42.57	±0.91
TEA_TCPP_30	38.10	±0.32	40.47	±0.30	42.30	±0.38
TEA_TEP_10	38.35	±0.09	40.44	±0.21	42.44	±0.17
TEA_TEP_20	38.56	±1.08	40.80	±1.15	42.38	±0.17
TEA_TEP_30	38.52	±1.19	40.63	±1.44	42.63	±1.85
TEA_DMPP_10	37.52	±0.14	39.11	±0.33	41.34	±0.29
TEA_DMPP_20	37.50	±0.22	39.35	±0.21	40.47	±1.08
TEA_DMPP_30	36.73	±0.81	38.46	±0.70	40.35	±0.69
DEG_REF	39.15	±0.44	41.34	±0.58	43.17	±0.44
DEG_TCPP_10	39.26	±0.44	41.22	±0.58	43.64	±1.30
DEG_TCPP_20	38.46	±0.05	39.97	±0.13	41.63	±0.18
DEG_TCPP_30	37.93	±0.09	39.86	±0.27	41.86	±0.46
DEG_TEP_10	38.39	±0.25	40.63	±0.36	42.63	±0.40
DEG_TEP_20	38.75	±1.25	41.07	±1.28	42.70	±1.09
DEG_TEP_30	38.54	±0.63	40.26	±0.72	41.85	±1.00
DEG_DMPP_10	38.64	±0.01	40.56	±0.37	41.90	±1.08
DEG_DMPP_20	36.13	±0.12	37.83	±0.04	39.90	±0.06
DEG_DMPP_30	35.19	±0.13	36.95	±0.13	38.91	±0.07

**Table 6:** Cone calorimeter results for TEA and DEG series modified with fire retardants.

Sample	TTI	TOF	pHRR	MARHE	THR	EHC	TSR	Residue [%]	LOI [%]
	[s]	[s]	[kW/m^2^]	[kW/m^2^]	[MJ/m^2^]	[MJ/kg]	[m^2^/m^2^]		
TEA_REF	2 ± 1	45 ± 2	297 ± 21	198 ± 11	7 ± 2	18 ± 2	277 ± 37	3 ± 1	19.6
TEA_TCPP_30	3 ± 1	44 ± 1	254 ± 11	169 ± 11	6 ± 0	17 ± 1	300 ± 25	6 ± 1	21.7
TEA_TEP_30	3 ± 1	45 ± 5	259 ± 2	179 ± 5	6 ± 1	17 ± 1	303 ± 38	12 ± 4	21.2
TEA_DMPP_30	5 ± 2	50 ± 13	181 ± 41	135 ± 35	6 ± 1	17 ± 1	284 ± 40	11 ± 4	22.0
DEG_REF	3 ± 1	46 ± 6	263 ± 25	201 ± 18	7 ± 0	21 ± 1	255 ± 18	13 ± 4	18.6
DEG_TCPP_30	3 ± 1	43 ± 4	227 ± 15	161 ± 7	6 ± 0	16 ± 1	307 ± 24	11 ± 3	21.3
DEG_TEP_30	2 ± 1	51 ± 4	232 ± 20	170 ± 11	7 ± 0	19 ± 1	313 ± 19	12 ± 3	21.0
DEG_DMPP_30	3 ± 1	54 ± 3	182 ± 2	144 ± 12	6 ± 0	17 ± 4	316 ± 29	16 ± 4	21.8

**Table 7:** TGA results of TEA and DEG series.

Symbol	T_d 5%_ [°C]	T_d 10%_ [°C]	T_d max1_ [°C]	T_d max2_ [°C]	T_d max3_ [°C]	Char at 750 °C [wt.%]
TEA_REF	275	301	-	360	470	14.7
TEA_TCPP_30	207	262	209	349	477	14.0
TEA_TEP_30	234	281	171	339	440	16.4
TEA_DMPP_30	234	278	167	354	427	22.5
DEG_REF	274	292	-	336	408	16.9
DEG_TCPP_30	205	268	190	333	409	16.5
DEG_TEP_30	208	275	154	339	407	19.1
DEG_DMPP_30	259	278	147	325	389	18.1
